# New Structural and Functional Contexts of the Dx[DN]xDG Linear Motif: Insights into Evolution of Calcium-Binding Proteins

**DOI:** 10.1371/journal.pone.0021507

**Published:** 2011-06-24

**Authors:** Daniel J. Rigden, Duncan D. Woodhead, Prudence W. H. Wong, Michael Y. Galperin

**Affiliations:** 1 Institute of Integrative Biology, University of Liverpool, Liverpool, United Kingdom; 2 Department of Computer Science, University of Liverpool, Liverpool, United Kingdom; 3 National Center for Biotechnology Information, National Library of Medicine, National Institutes of Health, Bethesda, Maryland, United States of America; King Abdullah University of Science and Technology, Saudi Arabia

## Abstract

Binding of calcium ions (Ca^2+^) to proteins can have profound effects on their structure and function. Common roles of calcium binding include structure stabilization and regulation of activity. It is known that diverse families – EF-hands being one of at least twelve – use a Dx[DN]xDG linear motif to bind calcium in near-identical fashion. Here, four novel structural contexts for the motif are described. Existing experimental data for one of them, a thermophilic archaeal subtilisin, demonstrate for the first time a role for Dx[DN]xDG-bound calcium in protein folding. An integrin-like embedding of the motif in the blade of a β-propeller fold – here named the calcium blade – is discovered in structures of bacterial and fungal proteins. Furthermore, sensitive database searches suggest a common origin for the calcium blade in β-propeller structures of different sizes and a pan-kingdom distribution of these proteins. Factors favouring the multiple convergent evolution of the motif appear to include its general Asp-richness, the regular spacing of the Asp residues and the fact that change of Asp into Gly and vice versa can occur though a single nucleotide change. Among the known structural contexts for the Dx[DN]xDG motif, only the calcium blade and the EF-hand are currently found intracellularly in large numbers, perhaps because the higher extracellular concentration of Ca^2+^ allows for easier fixing of newly evolved motifs that have acquired useful functions. The analysis presented here will inform ongoing efforts toward prediction of similar calcium-binding motifs from sequence information alone.

## Introduction

Calcium-binding proteins (CaBPs) regulate a variety of cellular processes, including cell division, differentiation, motility and apoptosis [1]–[3]. In addition, Ca^2+^ ions serve as cofactors in a number of (mostly hydrolytic) enzymes [4]. Sequence and structural comparisons identified a number of different Ca^2+^-binding sites [5]–[8] that coordinate Ca^2+^ ions with 6 or 7 coordination bonds [6]. The best known Ca^2+^-binding motif is a helix-loop-helix structure, referred to as the EF-hand [9]–[12]. In the canonical EF-hands, Ca^2+^ ions are coordinated by oxygen atoms from the side chains of the first, third, and fifth residues from the loop (which are usually Asp residues - the third and, less frequently, the fifth residue can alternatively be Asn). Additional coordination bonds are provided by the backbone oxygen atom of the seventh loop residue (which can be any residue), a water molecule coordinated by the side chain of the ninth loop residue (which is usually D, E, S, T or N), and the side chain of an acidic (usually Glu) residue in the 12^th^ position from the beginning, which is typically located at the start of the second helix [9], [10], [12], [13]. Additional conserved residues include Gly in the sixth position and a hydrophobic residue (Ile, Leu or Val) in the eighth position of the loop [14]. As a result, the first 10 residues of the Ca^2+^-binding loop of the EF-hands structure typically form a Dx[DN]xDGx[ILV][DSTN]x sequence pattern, see [15].

We have previously studied the distribution of the DxDxDG-containing loop among proteins of known structure and found this loop in an impressive variety of non-EF-hand structural contexts [15]–[17]. In contrast to the helix-loop-helix EF-hand structure, these included helix-loop-strand, helix-loop-turn, strand-loop-helix, strand-loop-strand, and several structural contexts without a regular secondary structure element either before or after the DxDxDG-containing loop [15]. In each of these cases the loops demonstrably bound Ca^2+^ ions and the calcium-binding ligands superimposed extremely well. Furthermore, insertion of such a DxDxDG-containing, Ca^2+^-binding loop between two β-strands of rat CD2 protein proved sufficient to create a new Ca^2+^-binding site [18], [19].

These data clearly demonstrated that the DxDxDG-containing Ca^2+^-binding loop was a separate well-defined structural element and raised the question as to how it arose in such similar forms in so many unrelated protein folds. Two hypotheses were put forward to explain the diversity of the DxDxDG-containing calcium-binding loops: 1) a putative novel mechanism involving transplant of 10–12 residue Ca^2+^-binding loops between different protein contexts or 2) local convergent evolution within an existing loop structure leading to the emergence of the DxDxDG motif [15].

Here we report and analyse further instances of the Ca^2+^-binding DxDxDG loop revealed by rapidly expanding knowledge of the protein structure universe. Given sequence trends at the third position, not only in EF-hands but also in the novel examples, we introduce here the Dx[DN]xDG name, although it must be noted that as a strict regular expression, Dx[DN]xDG covers most but not all of the calcium-binding motifs characterized here. We further consider the evolutionary mechanisms that are responsible for the origin and maintenance of the Ca^2+^-binding sites. The results have important implications for the prediction and interpretation of similar motifs in protein sequence databases.

## Results and Discussion

### General description

**Figure 1:**
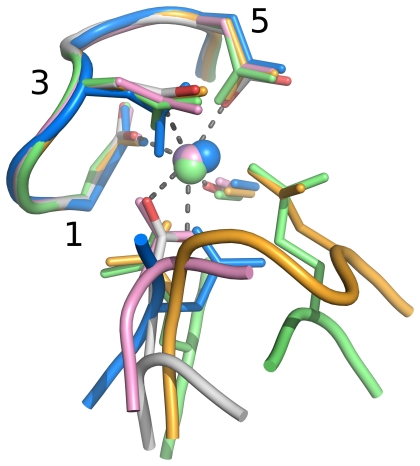
Comparison of Dx[DN]xDG calcium-binding motifs in calmodulin and the new structural contexts presented here. The metal (sphere) is bound by the side chains of the Dx[DN]xDG motif (labelled 1, 3, 5) and the carbonyl group of the residue immediately following the motif. These, and the entire motif backbone, superimpose very well, while additional contributions to binding from later residues vary hugely in spacing and number (see text, Table 1 and Fig. 2). The representative calmodulin (PDB code 1exr) is coloured by atom type, with carbon white, oxygen red and bound calcium in purple. Other structures and their bound calcium ions are coloured uniformly with *T. kodakaraensis* subtilisin (PDB code 2z2x) in orange, endo-α-N-acetylgalactosaminidase (PDB code 2zxq) in pink, *E. coli* YgjK (PDB code 3c68) in green and the *Porphyromonas* adhesion domain (PDB code 3km5) in blue. Interactions of calmodulin with bound metal are shown as dotted lines.

**Figure 2:**
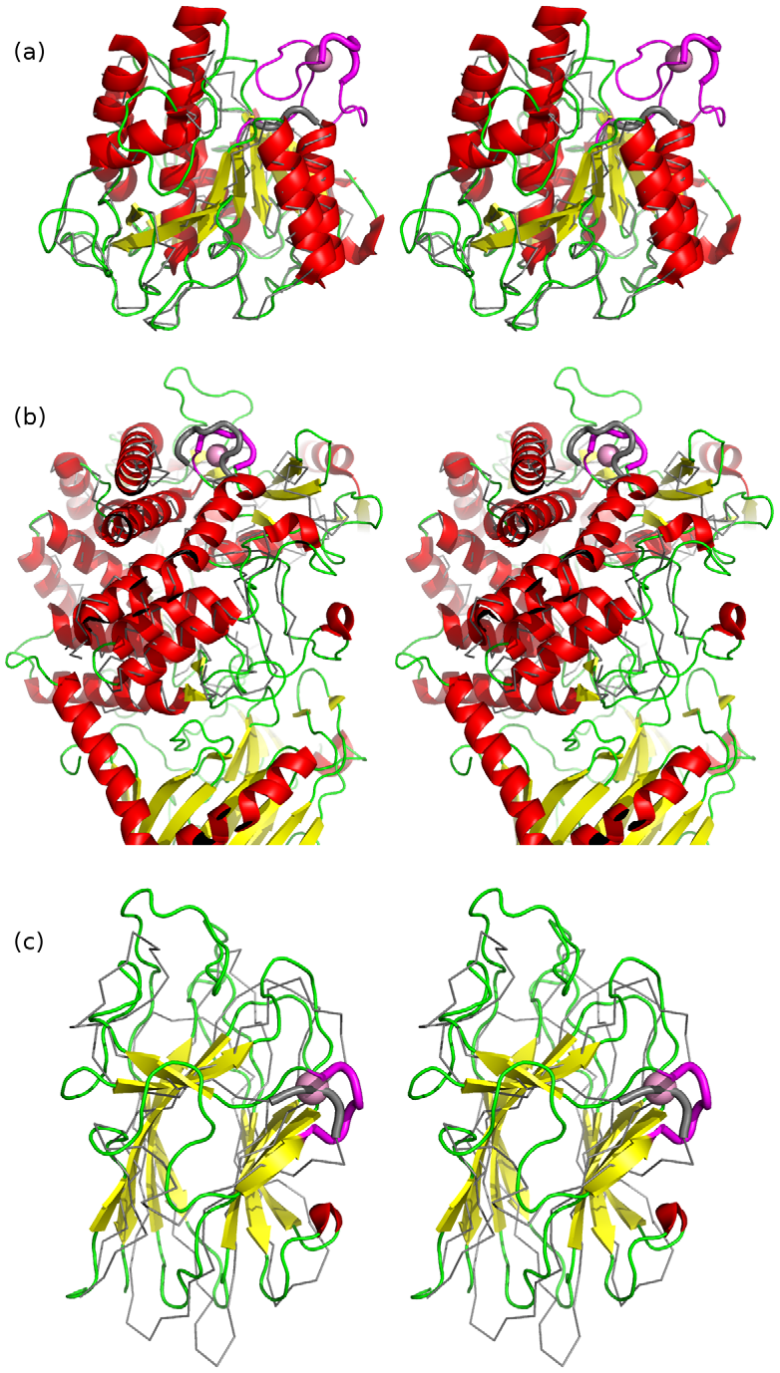
Stereo structure superpositions of novel Dx[DN]xDG calcium-binding motifs with nearest non-calcium binding structural neighbours. Panel a) shows *T. kodakaraensis* subtilisin (PDB code 2z2x), b) *E. coli* YgjK (PDB code 3c68) and c) the *Porphyromonas* adhesion domain (PDB code 3km5). In each case the Dx[DN]xDG motif is shown as a thick magenta cartoon with bound calcium in pink and the remainder of the calcium binding protein coloured by secondary structure. In a) the Dx[DN]xDG motif is positioned in a larger insertion binding four calcium ions which is also shown in magenta. Structural neighbours (*Bacillus lentus* subtilisin (PDB code 1c9m) in a), a predicted hydrolase from *Thermus thermophilus* (PDB code 2z07) in b), and an adhesion domain from human Tyr phosphatase mu (PDB code 2v5y) in c) are in grey with the portion aligning to the calcium binding region shown as thick cartoon. Note that the fourth novel context (2zxq) has no non-calcium binding structural neighbour in the present PDB.

The new data presented here show four entirely new folds to harbour Dx[DN]xDG calcium-binding loops that superimpose very closely on the archetypal EF-hand motifs (Table 1, Figs. 1 and 2). These new folds are all-α (the α/α toroid of *E. coli* glycoside hydrolase YgjK [20]), all-β (the supersandwich of a glycoside hydrolase from *Bifidobacterium longum* [21]; and the galactose-binding domain-like fold of a *Porphyromonas* adhesin [22]) or mixed α+β (*Thermococcus* subtilisin [23]). The similarities between these calcium-binding loops and those of EF-hands or other instances of the Dx[DN]xDG motifs have not been reported previously. These new examples significantly expand the range of the Dx[DN]xDG motifs, currently visible in 16 different structural contexts. Yet more examples may await discovery.

**Table 1:** Novel families containing Dx[DN]xDG calcium-binding loops.

Representative^a^^,^ b	SCOP class; fold of domain containing Dx[DN]xDG loop	PDB code, reference; position of the first D of Dx[DN]xDG	Distribution of proteins containing Dx[DN]xDG loop	Frequency of the Dx[DN]xDG loop in		R.m.s. fit of Dx[DN]xDG to the first calmodulin motif	Distance between Dx[DN]xDG and later Ca^2+^ ligands (aa)	Function of bound calcium	Broader molecular function, shared by Ca^2+^-binding proteins and non-binding homologues
				homologous proteins^c^	phhmer neighbors^d^				
**Novel structural contexts**									
*Thermococcus kodakaraensis* subtilisin	α+β; Subtilisin-like^d^	2z2x [92]; 212	Some thermophilic archaea	<1% (Pfam PF00082)	<1%	0.12	4/7	Folding, in the context of a 25-residue insertion [23]	Proteolysis
*Bifidobacterium longum* endo-α-N-acetylgalactosaminidase	All β; Supersandwich^e^	2zxq [21];601	Bifidobacteria	Approx 7%	34%	0.15	5	Possibly structural [21]	Carbohydrate digestion
*Escherichia coli* YgjK, glycoside hydrolase family GH65	All α; α/α-toroid^e^	3c68 [20]; 431	Some gamma-proteobacteria	Approx 7% (Pfam PF01204)	37%	0.12	2/112	Not known	Carbohydrate digestion
*Porphyromonas gingivalis* gingipain adhesin domain	All β; Galactose-binding domain-like^e^	3km5 [22]; 1179	*Porphyromonas*, *Flavobacterium*	Approx 90% (Pfam PF07675)	83%	0.35	36	Possibly structural [22]	Carbohydrate binding
**Calcium blades**									
Human integrin αVβ3	All β; 7-bladed β-propeller	1jv2 [93]; 284; 349; 413	Eukaryotes	100% of 3 sites (Pfam PF01839)	90%, 100%, 94%	0.61–1.07	2	Potentially regulatory [90]	No shared broad molecular function
*Bacillus subtilis* rhamnogalacturonan lyase	All β; 8-bladed β-propeller^e^	2z8r [24]; 158; 222; 369	g	g	100%, 100%, 96%	0.22–0.36	2	Not known (further calcium-binding site required for activity) [24]	
*Pseudomonas aeruginosa* pilus biogenesis factor PilY1	All β; 7-bladed β-propeller^e^	3hx6 [25]; 851	Bacteria	Approx 75% (Pfam PF05567)	100%	0.37	2	Regulation of pilus biogenesis and motility [25]	
*Psathyrella velutina* lectin	All β; 7-bladed β-propeller^e^	2bwr [26]; 177; 233; 343^f^	g	g	100%, 81%, 95%	0.48–0.55	2	Possibly structural [26]	

a A version that includes previously reported families is provided as Table S1.

b All these proteins have been experimentally demonstrated to bind calcium ions.

c As defined by Pfam, SMART or by full-length matches in PSI-BLAST (E-value of 0.0001) run until convergence.

d Proteins from UniRef90 with e-value<0.001. See Methods for details.

e Based on the entry for a homologous protein or the authors' description.

f The motif commencing residue 233 is not bound to calcium in the deposited structure but crystal soak data show that it is capable of doing so [26].

g A distinct group could not be defined with PSI-BLAST.

We previously noted the Dx[DN]xDG motif in the extracellular β-propeller of integrin. Here we report similar motifs in differently sized propeller domains of two bacterial proteins, *Bacillus subtilis* rhamnogalacturonan lyase [24] and *Pseudomonas aeruginosa* PilY1 [25] and a fungal lectin [26]. The resemblance of the motif of the last to the EF-hand has not previously been noted. The relationships between and distributions of the propeller-borne motifs, here named calcium blades, are considered later. Asn is present at the third position of the motif with a frequency approaching that of Asp, hence the change in nomenclature from the DxDxDG to the Dx[DN]xDG motif.

For the newly described structures, calcium binding is crystallographically observed in all cases except for the *Bifidobacterium* endo-β-N-acetylgalactosaminidase. In that crystal structure manganese is bound to the Dx[DN]xDG motif but calcium may be considered as a stronger candidate for *in vivo* binding due to its much higher concentration in the environment. Calcium is bound at this position in the homologous (48% sequence identity) enzyme from *Streptococcus pneumoniae* [27]. Confirmed calcium-binding proteins such as EF-hands have been crystallized in complex with a variety of metals including manganese.

The newly discovered motif examples recapitulate the remarkable local structural homogeneity in the vicinity of the motif (Fig. 1; Table 1). This was assessed quantitatively through measuring root mean square deviations (RMSD) of corresponding atoms following superposition of the new six amino-acid motifs on the first EF-hand of *Paramecium tetraurelia* calmodulin (PDB code 1exr [28]), this latter employed as a reference. Since the amino acids varied, detailed side chain comparisons were not possible and the measurements were based on ‘extended main chain atoms’ (i.e. main chain N+Cα+C+O plus Cβ - virtual Cβ in the case of Gly). The resulting RMSD values were no more than 0.55 Å indicating that the new motifs superimposed extremely well on this reference EF-hand structure. For comparison, the other calcium-binding motifs in *Paramecium tetraurelia* calmodulin yield RMSD values of up to 0.42 Å.

In each of the new motif examples, the backbone carbonyl of the residue immediately following the motif contributes to metal binding (Fig. 1). As before, Asp residues, with occasional substitution by Asn, predominate at the D positions of the motif, justifying the continued use of the name. However, an interesting novelty is present in the *Psathyrella velutina* lectin structure [26]) where the second D position is occupied by Thr. This residue was not previously observed in one of the key positions of the motif, although Ser was twice seen at the second D position in our earlier examples [15]. Inspection of the crystal structures shows that both the Ser and Thr residues ligate the metal through lone pairs on their side chain oxygen atoms. For example, the separation of the Oγ1 atom Thr345 and bound calcium in lectin structure is 2.4 Å, a figure that may be compared to a typical calcium-H_2_O interaction distance of 2.39 Å [29].

**Figure 3:**
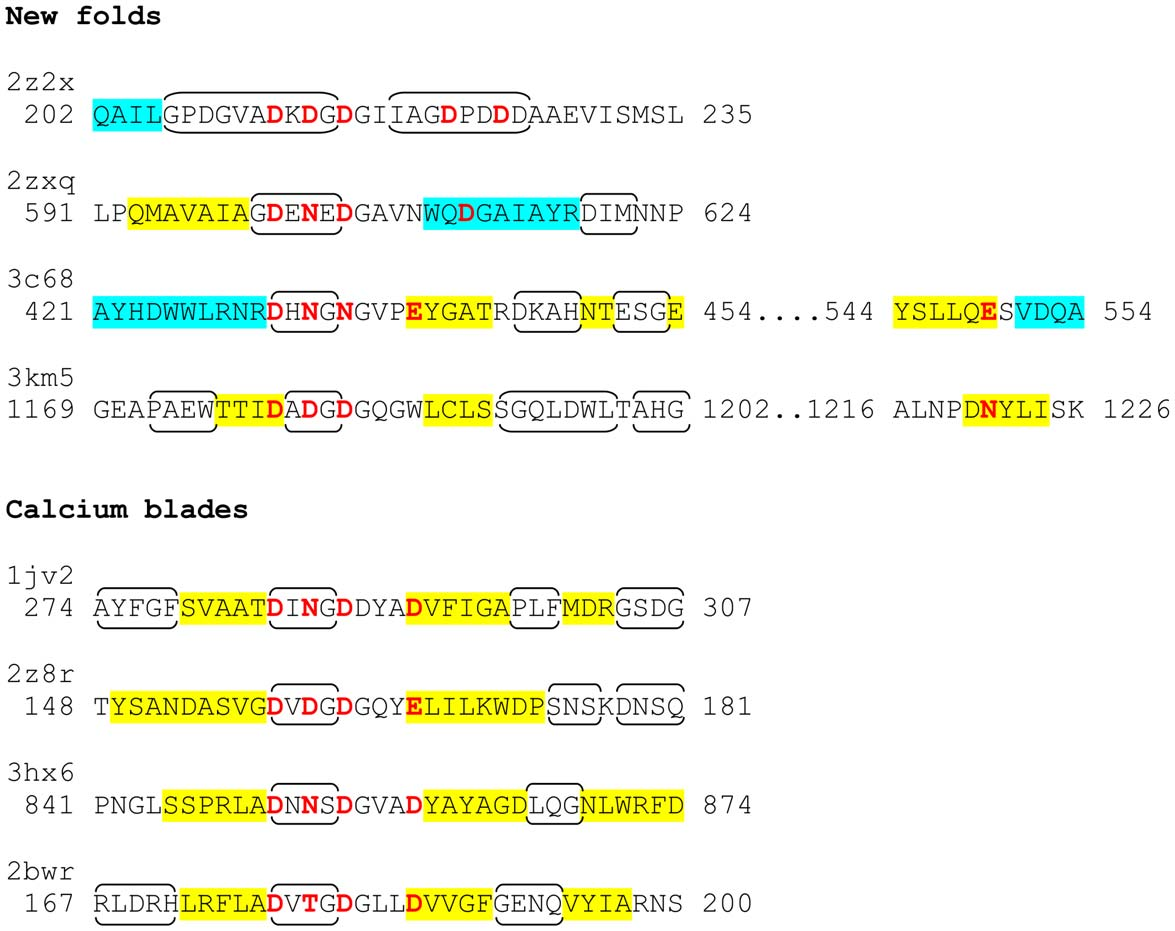
Secondary structure context of the Dx[DN]xDG motifs, highlighting additional metal-binding residues (**Table 1**). Residues binding to metal using side chains are in red (direct interaction with calcium) or purple (through-water interaction). Secondary structure as defined by STRIDE [78] is indicated as follows: α-helices, blue shading; β-strands, yellow shading; turns, brackets. A version including previously reported families is included as Figure S1.

As previously, the side chain interactions from the D positions and the main chain interaction with the bound Ca^2+^ ions are supplemented by the interaction of side chains from at least one further acidic residue (or, occasionally an amide residue). Remarkably, all the new examples follow precedent in positioning the additional residue(s) later in the protein sequence: in not a single example from 16 different folds positions does the additional residue occur before the motif. We previously observed striking variation in the separation of the Dx[DN]xDG motif and the additional residue, from a minimum of two intervening residues to a maximum of 65. With the exception of the *Bifidobacterium* glycoside hydrolase, which has a separation of 5 residues, the new examples presented here have hitherto unseen separations of 4, 7, 36 and 112 residues (Table 1, Fig. 3). Curiously, naturally observed binding geometries do not, so far, include that of the artificially engineered EF-hand variant which was designed to include direct side chain interactions by residues separated by 2 or 5 residues, respectively, from the Dx[DN]xDG motif [30].

Most of the new examples conform to the previously common pattern in which the Dx[DN]xDG motif is positioned in a loop flanked by elements of regular secondary structure (Fig. 3). As before, the upstream and downstream secondary structures may equally well be β-strands or α-helices. The exception to this trend is the subtilisin structure in which the Dx[DN]xDG motif is part of a 25-residue, irregular insertion into the subtilisin fold that is stabilised by binding of four Ca^2+^ ions.

We previously discovered homologous binuclear calcium-binding motifs involving Dx[DN]xDG sequences in anthrax protective antigen (PDB code 1acc [31]) and human thrombospondin (PDB code 1ux6 [32]). One of the new structures, that of *Thermococcus* subtilisin shows a different kind of binuclear centre in which the second and third D positions of the Dx[DN]xDG motif, and one of the two additional residues contribute to the binding of a second Ca^2+^ ion. A further Asp residue, exclusive to the second site, completes the binding. When the Dx[DN]xDG motifs of subtilisin and thrombospondin are superimposed, the second calcium ions also superimpose perfectly, yet the differences elsewhere, including the fact that two more calcium ions are bound nearby in subtilisin, show that the subtilisin binuclear site is not homologous to the others.

The sequence conservation of the motifs was assessed in two ways. Motif conservation was first measured in the set of proteins retrieved in a simple database search with phmmer [33], [34] (see Methods and Table 1). This shows the motifs in calcium blades (see below) to be well conserved but, in contrast, the motif to be present in only a tiny fraction of subtilisin-like sequences. Other motif instances exhibit intermediate conservation. Motif frequency was also assessed with respect to Pfam families or, where unavailable, the results of iterative database searches (Table 1). The frequency of predicted functional motifs tends to be lower in these sets of broader homologues, as expected. For example, the motif in *Escherichia coli* YgjK is conserved in functional form in 37% of phmmer homologues but in only 7% of the large trehalase Pfam family (PF01204).

When compared with the previous set of Dx[DN]xDG structural contexts, the new examples are generally of narrower phyletic distribution. The most extreme example is that of the gingipain adhesion domain where, in the current sequence databases, the Dx[DN]xDG motif is confined to *Porphyromonas gingivalis*. This may reflect the increasingly complete coverage of large pan-phyla families in the PDB, at least among soluble proteins. Among our previous set of motifs, instances in archaea were rather rare, being confined to a few EF-hands and dockerin domains plausibly originating from lateral gene transfers. It is interesting, therefore, to see in the new results an archaea-specific Dx[DN]xDG motif found in a few thermophiles. This suggests that there may not be an intrinsic bias against evolution of the motif in archaea, rather a simple under-representation of their sequences in the current databases.

Interestingly, it has become increasingly clear that known examples of the Dx[DN]xDG motif have a strong bias towards periplasmic or cell surface localization or secretion. The only proven exceptions so far appear to be the EF-hands, an isolated member of the transglutaminase family [35] and some calcium blades (see below). This may reflect the fact the extracellular concentrations of calcium are much higher than generally found inside cells [36], [37] so that newly generated motifs are ‘fixed’ more often in the extracellular milieu through acquisition of useful functions.

### Propeller-borne Dx[DN]xDG motifs: the calcium blades

**Figure 4:**
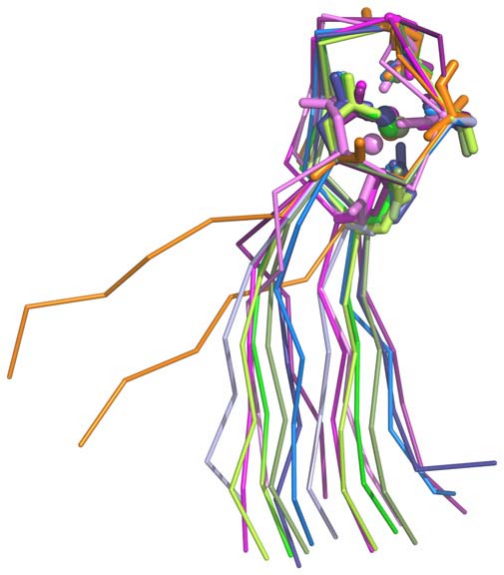
Comparison of calcium blades and their flanking β-strands. Backbone is shown as ribbon, side chains that interact with metal as sticks and the metal ions as small spheres. The structures are coloured as follows: integrin (PDB code 1jv2; three examples) in shades of pink, lectin (2bwr; three examples) in shades of green, rhamnogalacturonan lyase (2z8r; three examples) in shades of blue and PilY1 (3hx6) in orange.

Remarkably, as Table 1 shows, there are now four distinct examples in the PDB of calcium-binding Dx[DN]xDG motifs found at the tips of the blades of β-propeller folds. First seen in integrin [15], they are now also visible in two bacterial proteins and in a fungal lectin. This immediately raises the question of whether the four instances share a common evolutionary origin. As Fig. 4 shows, metal binding geometries in the four proteins are very similar and in each case the separation of motif and additional side chain interaction is two residues (Fig. 3). The orientation of the motif with respect to its flanking β-strands is similar for all cases except PilY1 but the difference in the latter still appears compatible with a shared common origin of them all. Equally, the fact that the propellers differ in the number of blades – seven except for the eight in rhamnogalacturonan lyase – is not strong evidence against homology since it is known that propellers can readily evolve through duplication of an entire blade [38].

**Figure 5:**
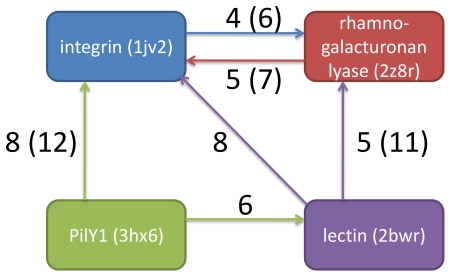
Schematic representation of statistically significant relationships between calcium blades revealed by JackHMMER [**39**] iterative database searches. Arrows indicate retrieval of a given motif by a query, with numbers indicating the number of iterations required at e-values of 0.01 or, bracketed, 0.001.

Using the modern, sensitive database searches of the HMMER3 package [33], [34], connections between the four calcium blades are readily demonstrated. We took the region comprising the motif and downstream additional residue – Dx[DN]xDG–[D/E] – along with six flanking residues both before and after. Database searches with the JackHMMER program [39] in the nr protein database [40] of up to 30 iterations were carried out using e-values of either 0.01 or 0.001. As Fig. 5 shows, even at the more stringent e-value the Dx[DN]xDG motifs of the four different propellers could be connected by statistically significant relationships. Importantly, at e = 0.001, the search results were uncontaminated by non-propeller instances of the Dx[DN]xDG motifs. At the more permissive e = 0.01, EF-hands were occasionally picked up by the searches, but were inevitably discarded in later iterations and therefore absent from the final results.

Importantly, the likely homology of calcium blades is not evident from browsing current domain databases. Integrin is represented by the FG-GAP (PF01839) or Int_alpha (SM00191) domains in Pfam and SMART, respectively, both of which entries inform that some members contain a calcium-binding site. The fungal lectin and rhamnogalacturonan lyase match no domains with default search parameters, although raising the e-value cut-off produces weak matches to the FG-GAP domain. The PilY1 protein matches the Neisseria_PilC entry in Pfam (PF05567) with no indication of a propeller fold.

Since the FG-GAP and Int_alpha domain entries span whole propeller blades and contain many blades that lack Dx[DN]xDG motifs it appears that the calcium blades map awkwardly onto present domain databases, only being present in a subset of FG-GAP matching regions, but simultaneously existing in proteins not matching the FG-GAP domain. This prompted us to search for further instances of this type of Dx[DN]xDG motif in the human genome. Using the results of the iterative database searches described above various integrins and integrin-like proteins were retrieved, as expected, along with the related domains known to be present in phosphatidylinositol -specific phospholipase D [41] and the motifs recently described in cartilage acidic protein [42]. Three novel proteins containing two motifs each (Table 2) were also recovered with significant e-values; proteins that were independently confirmed to be β-propellers by profile-profile matching. These examples are poorly visible in databases – UniProt entries reveal just a single FG-GAP domain in T cell immunomodulatory protein (TIP), while sequence searches at Pfam produce FG-GAP hits (three) for only kaptin. The secreted or cell-surface TIP has been characterised as an immunomodulatory protein that stimulates T-cells to secrete several cytokines [43]. The *Caenorhabditis elegans* orthologue of TIP is implicated, by RNAi experiments catalogued in WormBase [44], in reproduction, embryonic and larval development. Interestingly, a related protein in *Cryptococcus neoformans* that shares about 26% sequence identity with TIP, is a known virulence factor of that fungal pathogen [45]. Most intriguingly, kaptin and Bardet-Biedl syndrome 2 protein (BBS2) are both intracellular proteins in contrast to the exclusively extracellular calcium blades previously characterised. Kaptin is an actin-binding protein [46], [47] localized at the tips of stereocilia in cochlea [48], bodies related to the mechanotransduction of sound. This, and the location of its gene near a known deafness locus, strongly implicate the protein in audition [48]. BBS2 and another protein, BBS4, localise to cellular structures associated with motile cilia and which are required, not for their synthesis, but for the structural integrity and function of the mature cilia [49]. Interestingly, more recent data also implicate BBS proteins in signalling via the leptin receptor [50]. While the role of calcium binding to these proteins remains to be confirmed, it is probably significant that the D174E mutation in BBS2, which is in general a well-accepted substitution [51] but in this case predicted to abolish calcium binding to one of its motifs, is associated with the disease [52].

**Table 2:** Novel putative calcium blades in human proteins.

UniProt ID	Protein name	Length (residues)	Subcellular localization	Number of predicted binding motifs^a^	Motif sequences
BBS2_HUMAN	Bardet-Biedl syndrome 2 protein (BBS2)	721	Cytoplasm/cilium membrane [94]	2	170 DFDGDGKKE 178 b 251 DLNSDGVNE 259
KPTN_HUMAN	Kaptin	436	Cytoplasm/actin filaments [46], [47]	2	317 DVDLDGRPE 325 373 DLTGDGLQE 381
TIP_HUMAN	T cell immunomodulatory protein (TIP)	612	Extracellular and/or transmembrane [43]	2	266 DFDGDGHMD 274 338 DYNMDGYPD 346

a Estimated conservatively: substitutions at the key positions of the DxDxDG motif are only allowed if precedents exist in Figure 3.

b An Asp174→Glu mutation has been identified in a patient with Bardet-Biedl syndrome [52].

It is interesting to note the functional parallels between stereocilia and cilia with which kaptin and BBS2, respectively, are associated, particularly since the relationship between the two proteins, in statistical terms, is at best borderline significant. For example, bending of both stereocilia and cilia results in entry of calcium into the cell through ion channels [53], [54]. More broadly, it is perhaps more than coincidence that bacterial PilY also contains a calcium blade: historically, the homology between BBS8 and the bacterial PilF protein, involved in pilus assembly and twitching, provided an initial clue that Bardet-Biedl syndrome could be related to defects in cilia function [55].

Elsewhere, the distribution and abundance of calcium blades seems to vary widely. Model organisms *Escherichia coli* and *Saccharomyces cerevisiae* lack the motif entirely, but it is present in some archaea, in two proteins from *Methanosarcina acetivorans* and one from *Archaeoglobus fulgidus*, but not in *Sulfolobus solfataricus*. The ease with which propeller blades duplicate [38] and structural plasticity of the results [56] are probably responsible for some spectacular tandem duplications of the motif evident in sequence databases. Currently, the most extensive is a protein coded by locus Npun_R4253 in the cyanobacterium *Nostoc punctiforme* in which there appear to be three tandem, seven-bladed propellers formed largely of calcium blades.

### Function of the new Dx[DN]xDG motifs

Broadly speaking, functions of our previously reported set of Dx[DN]xDG motif proteins could be divided into structural or regulatory roles. In the former, an essentially permanent metal interaction with protein was considered to stabilise the protein fold. In contrast, regulatory roles involve variation in the calcium binding status of the protein according to prevailing local calcium concentration with functional implications. Among the new structural contexts (Table 1) the literature shows that structural functions of bound calcium have been tentatively proposed in two cases. More interestingly, experimental data indicate a novel function for bound calcium in the case of *Thermococcus kodakaraensis* subtilisin (Tk-subtilisin): an essential role in the folding of the protein. Subtilisins are of interest as model systems for studying the thermodynamics and kinetics of protein folding since the final structure of the mature protein strongly depends on the propeptide portion ([56]). Unusually, and in contrast to bacterial subtilisins, Tk-subtilisin requires calcium for proper folding, even in the presence of its propeptide sequence [57] which, atypically, is not required for folding [58]. This calcium requirement has been assigned to the four-calcium insertion containing the Dx[DN]xDG motif [58]: an insertion-less mutant failed to fold. An attempt was made to specifically eliminate the Dx[DN]xDG calcium site: the mutant could fold, but interpretation of the role of the bound calcium was complicated by compensatory structural changes [59]. While folding requires the whole insertion, with its four calcium sites, this is still the first clear example of the involvement of Dx[DN]xDG-bound calcium in the protein folding process. Earlier data on mutants of glycosylphosphatidylinositol-specific phospholipase D with reduced metal binding to its propeller-borne Dx[DN]xDG sites showed dramatically reduced expression. An effect on protein folding would be one explanation, but the reduction could equally well result from impaired intracellular transport or secretion [60].

As mentioned above, a single substitution in one of the propeller-type motifs in BBS2 is enough to lead to disease suggesting that calcium plays an important role in its function. Experimental data also clearly show the importance of calcium binding to the related motifs in PilY1 protein [25]. Chelation of calcium or mutation of the Dx[DN]xDG motif each leads to loss of *Pseudomonas* twitching motility through elimination of surface pili. Surprisingly, the functions of the propeller-bound calcium ions in integrin remain mysterious [61]. Nevertheless, although not all Dx[DN]xDG motifs have been experimentally probed, it is already apparent that at least a large proportion of these motifs have structural and/or functional importance to their respective proteins.

Very recently, structural and dynamic analysis of metal-binding proteins has demonstrated their particular suitability for signal propagation, a property possibly related to the relative rigidity of the sites themselves [62]. This finding may go some way to explain the frequency with which signalling and regulatory functions are associated with Dx[DN]xDG motif calcium-binding proteins (Table 1 and Table S1).

### Evolution of Dx[DN]xDG motifs

We previously argued that the unrelated structural contexts in which superimposable motifs were found implied their arising by either an as-yet uncharacterised splicing of loops from one protein to another, or multiple convergent evolution. Since then the awareness of the scientific community of the power of convergent evolution has increased significantly. Not only do enzymes exhibit convergently evolved mechanism but, more relevant to the present work, large numbers of convergently evolved linear motifs have been characterised, methods for their prediction produced [63], [64] and a database set up [65]. In the light of this literature, it appears that convergent evolution is the more likely explanation for the Dx[DN]xDG motifs, but the question still arises as to why it has evolved so frequently. In order to assess this frequency in comparison to other linear motifs, we examined the number of unrelated proteins known to contain examples of other motifs in a benchmarking subset of the ELM database [65] (see Table 1 of [64]). The mean number of motif instances in unrelated proteins for this set of 17 motifs was 9.2, but this value falls to 7.8 for motifs with four defined positions. Summing the present data with previously characterised Dx[DN]xDG motifs (see Table S1) produces at least 16 instances in unrelated proteins. Clearly, the Dx[DN]xDG motif has evolved more often than most well-characterised linear motifs.

As we have previously shown, there are many examples where homologous proteins differ in possession of the Dx[DN]xDG motif: one protein has a short motif-less loop between secondary structure elements while in a related protein a longer loop harbours a functional motif. Such differences in length can arise from various sources including slipping during replication resulting in single or double amino-acid repeats [66] or meiotic recombination events that can produce larger repeats [67].

Two characteristics of the Dx[DN]xDG sequence may facilitate its formation: its sequence bias, being Asp-rich, and its regularity. The possible contributions of each are now explained. The Dx[DN]xDG motif typically contains two or three Asp residues and, furthermore, the additional interactions required for metal binding may be provided by another Asp separated from the motif by as few as two residues. Clearly, generally acidic regions will be predisposed to form the motif, particularly as Glu may provide the later interaction. Thus, slippage mechanisms generating tandem single amino acid repeats [68], in this case of Asp residues, could be part of the explanation of the frequency of Dx[DN]xDG motif appearance. An interesting parallel can be drawn with the DxxDxxxD motif, convergently evolved multiple times for binding in partners of yeast protein phosphatase 1 [63]. As examination of Fig. 4 of Neduva et al. [63] illustrates, in that case as well many of the functional motifs evolved in generally acidic regions. It is also worth noting that seven out of the nine residues forming a different recently-described mode of calcium binding, the calcium bowl [69], are Asp residues although only two of their side chains interact with the metal.

A second notable characteristic is the regular nature of the motif: (Dx)_3_. In many instances of the motif one or other of the x positions, particularly the second, is occupied by Gly (Fig. 3). For example, in the *Porphyromonas* lectin, the motif sequence is DADGDG while in *Thermotoga maritima* 4-α-glucanotransferase it is DGDLDG. Thus, the slipping mechanism for repeat expansion, operating on a hexanucleotide sequence, could easily generate a nascent motif from a single instance of DG. Again, other comparable examples exist: methylated (RG)_n_ repeats bind to the Tudor domain [70] while (RS)_n_ motifs are common in the RS domains of SR (serine/arginine-rich) proteins and function in protein-protein interactions [71].

Finally, we note that only single nucleotide changes, of the more common transition type, separate Gly (coded by GGN in the genetic code) and Asp (GAT or GAC). This could ease the introduction of Gly into Asp-rich tracts or vice versa. Curiously, a single mutation, albeit a less common transversion, also separates Arg (AGA or AGG) and Ser (AGC or AGT), the components of the RS domain repeat mentioned above. Taken together, it seems likely that the biased composition – Asp richness – and regularity of the motif, along with the coding proximity of Asp and Gly, are at least significantly responsible for the anomalous frequency of the Dx[DN]xDG motif. Naturally, not every evolved Dx[DN]xDG motif will be structurally capable of adopting the characteristic metal-binding conformation. However, two factors may increase the proportion of Dx[DN]xDG motifs that are. First, the motif is indifferent to varied or absent flanking secondary structure, appearing simply to require a suitable structural separation of its beginning and end. Secondly, the additional residues required for metal interaction – acidic or amide group (Fig. 3) – are naturally abundant at the protein surface.

If the modes of evolution proposed above indeed played a role in producing the present day set of convergently evolved Dx[DN]xDG motifs then sequences resembling ancestral evolutionary intermediates might be present in current sequence databases. We therefore looked at motif presence or absence in the context of sequence clustering trees. Unfortunately, several factors conspired to limit the usefulness of the analysis including the fact that motifs in families of sequences tend to be either rare eg subtilisin or near universal eg the gingipain adhesion domain (Table 1). Furthermore, it is difficult to root trees composed of bacterial sequences, for example, given the lack of an external clock. Finally, the diversity of sequences in families led to a relative lack of well-supported nodes after bootstrapping analysis. Nevertheless, some features in well-supported structures of the tree derived from PilY1 (represented by PDB code 3hx6; see Table 1) and related proteins in Pfam family PF05567 (Fig S2) may shed light on modes of motif evolution. A group of four sequences from *Xanthomonas campestris* or *Stenotrophomonas* sp. SKA14 (marked with A in Fig S2) groups reliably with a set of *Xylella fastidiosa* sequences but lack the presumed functional motif DtDgDGlvD of the latter. Instead the four proteins have a longer Asp- and Gly-rich sequence such as DrwGGasqtDGvrDGyaD (in the protein with UniProt code Q4UW82). This may represent an Asp-rich, Gly-rich, ancestral-like protein or, alternatively, could be the relic of a motif inactivated by insertion. Another, acidic-rich, Gly-rich sequence positioned correspondingly to functional motifs elsewhere is found in a *Desulfuromonas acetoxidans* protein (Q1JW99; B in Fig S2) – DDGaGEk. Again, unfortunately, it is not possible to determine whether this is ancestral-like or simply the degraded result of a mutated, previously functional motif. Finally, examples of proteins containing single DG units are found in distinct parts of the tree in proteins from *Herminiimonas arsenicoxydans* (A4G7L9; C in Fig S2) and *Legionella pneumophila* (Q5X7C3; D in Fig S2): it is possible these resemble an ancestral-like sequence from which the motif evolved by duplication as outlined above although, of course, other scenarios can be imagined. It may be that this kind of analysis will be more productive in future, larger sequence databases which would lead to more confidently structured trees.

### Conclusions

The new instances highlighted here reinforce how exceptional the Dx[DN]xDG calcium-binding motif is. We are aware of no other comparable motif that has apparently convergently evolved so many times: shared general themes of 3D interactions with metals and small molecules are common (e.g. [72], [73]), but not the near structural uniformity observed for this linear motif (Fig. 1). Furthermore, the Dx[DN]xDG motif, unlike so many functional linear motifs [63], does not appear in regions of intrinsic protein disorder: indeed, our approach depends on the determination of motif structure by crystallography. We have highlighted, for the first time, specific features that are likely to have facilitated the appearance of the Dx[DN]xDG motif in so many structural contexts: consideration of these features may be relevant to future motif prediction efforts. Efforts are underway to exploit sequence trends – both in specific amino-acids and in broader physicochemical characteristics - and other information, such as appearance and spacing of predicted secondary structure elements, for the prediction of functional Dx[DN]xDG motifs from sequence alone. Given the widening and deepening understanding of the roles of calcium-binding Dx[DN]xDG motifs, such a method could contribute significantly to genome annotation.

## Methods

In order to search for new structural contexts for calcium-binding DxDxDG loops, searches were done, as before [15], using SPASM 3.7.3 [74]. A minimal query using only the D positions of the first such motif of *Paramecium tetraurelia* calmodulin (PDB code 1exr, sequence DKDGD [28]) was employed. Position-specific allowed residues were used based on the typical composition of such motifs: Asp was required at the first D position, at the second any of Asp, Asn, Ser or Thr was allowed while only Asp or Asn could be present at the third position. SPASM matches motifs based on two pseudoatom positions per residue, one each representing main chain and side chain, respectively. A SPASM library file containing PDB structures available as at June 2010 was generated locally using the MKSPAZ utility (http://xray.bmc.uu.se/usf/) and searched. The results were visually screened for bound metal. All the metal-binding motif hits contained Gly at the G position of the motif and shared the typical main chain loop conformation (Figs. 1,3). LSQMAN [75] was used for local structural superpositions including quantitative comparison of newly discovered motifs with a reference structure, first EF-hand of *Paramecium tetraurelia* calmodulin (PDB code 1exr [28]). Since sequences varied RMSD measurements were based on ‘extended main chain atoms’ (i.e. main chain N+Cα+C+O plus Cβ - virtual Cβ in the case of Gly). SSM [76] and DALI [77] were employed for fold comparisons e. g. to compare Dx[DN]xDG loop-containing structures with their nearest non-calcium-binding structural neighbours. These latter searches were done on the respective servers (http://www.ebi.ac.uk/msd-srv/ssm/; http://ekhidna.biocenter.helsinki.fi/dali_server/) using default parameters. Structures were visualised and manipulated in PyMOL (http://www.pymol.org). STRIDE [78] was used for secondary structure assignment in order to examine the position of the Dx[DN]xDG loop with respect to nearby secondary structure elements. Structural classifications were browsed in the SCOP [79] database and sequence domains in Pfam [80] and SMART [81].

Programs of the HMMER3 suite (http://hmmer.org; [33], [34]) were used for iterative database searching (JackHMMER [39] in order to discover distant sequence homologues in the nr sequence databases [40]; up to 30 iterations with e-value 0.01 or 0.001 were allowed. Genome mining was done using the resulting Hidden Markov Models (hmmsearch; e-value 0.001). Genome databases were obtained from UniProt (human; [82]) or the NCBI [40]. Motif occurrence in near sequence neighbours was evaluated as follows. Homologous sequences in the UniRef90 database [83] were obtained with phmmer [33], [34] using an e-value cut-off of 0.001. The queries in these cases were the structural domains containing the motifs or, in the case of calcium blades, the strand-turn-strand sequence in which the motif was embedded. The results were aligned with MUSCLE [84] and the occurrence of functional motifs assessed by search for a motif of the form Dx[DNST]x[DN][GADN]xx[DE]using the ps_scan software [85]. In this motif definition, the separation of Dx[DN]xDG motif and later calcium-binding residue(s) was required to match that seen in the crystal structures (Table 1) with the exception of large separations (>30 residues) where the later acidic reside was omitted from the motif definition. Profile-profile matching was done with HHPRED [86] employing default parameters and searching PDB [87] and/or Pfam databases [80]. This was done to sensitively annotate the Pfam domain structure of predicted calcium blade-containing sequences and to provide independent support for their containing β-propeller folds. Sequence alignments were visualised and manipulated with Jalview 2 [88]. A bootstrapped, neighbour-joining tree for the members of Pfam family PF05567 (Figure S2) was produced with MEGA4 [89]–[91] in order to assess their evolutionary relationship. Presumably due to the internal symmetry of the propeller structure the Pfam entry contains a large number of partial alignments. The sequences in the family were realigned with MUSCLE [84] and truncated down to the portion common to most members. This corresponded to residues 724–875 of the *Pseudomonas aeruginosa* protein of known structure (Table 1) – approximately the last three blades of the propeller.

## Supporting Information

Figure S1 Secondary structure context of the Dx[DN]xDG motifs, highlighting additional metal-binding residues (Table 1). The figure includes those motifs described in [15], Rigden & Galperin (2004) The DxDxDG motif for calcium binding: Multiple structural contexts and implications for evolution. J Mol Biol 343(4): 971–984. Residues binding to metal using side chains are in red (direct interaction with calcium) or purple (through-water interaction). Secondary structure as defined by STRIDE [74] is indicated as follows: α-helices, blue shading; β-strands, yellow shading; 3_10_ helices, green shading; turns, brackets.
(PDF)

Figure S2 Bootstrapped, neighbour-joining tree made with MEGA4 [90] using sequences edited and realigned from Pfam entry PF05567. Nodes with less than 50% bootstrap support have been collapsed. Individual sequences and groups mentioned in the text are labelled as follows: A, PilY1 sequences from *Xanthomonas campestris* and *Stenotrophomonas* sp.; B, *Desulfuromonas acetoxidans* PilY1-like protein Dace_0383 (UniProt: Q1JW99); C, *Herminiimonas arsenicoxydans* protein HEAR2375 (UniProt: A4G7L9); D, *Legionella pneumophila* protein Lpp0682 (UniProt: Q5X7C3).
(PDF)

Table S1 Families containing Dx[DN]xDG calcium-binding loops, including those in [15].
(PDF)
