# Supplementary material for: New Structural and Functional Contexts of the Dx[DN]xDG Linear Motif: Insights into Evolution of Calcium-Binding Proteins
Source: PLoS One. 2011 Jun 24;6(6):e21507. doi: 10.1371/journal.pone.0021507 (PMC3123361; doi:10.1371/journal.pone.0021507)
Supplement: Figure S1 — Secondary structure context of the Dx[DN]xDG motifs, highlighting additional metal-binding residues (Table 1). The figure includes those motifs described in [15], Rigden & Galperin (2004) The DxDxDG motif for calcium binding: Multiple structural contexts and implications for evolution. J Mol Biol 343(4): 971–984. Residues binding to metal using side chains are in red (direct interaction with calcium) or purple (through-water interaction). Secondary structure as defined by STRIDE [74] is indicated as follows: α-helices, blue shading; β-strands, yellow shading; 310 helices, green shading; turns, brackets. (PDF) [file pone.0021507.s001.pdf]

Figure S1. Secondary structure context of the Dx[DN]xDG motifs, highlighting additional metal-binding residues (Table 1). The figure includes those motifs described in ref. [15], Rigden & Galperin (2004) The DxDxDG motif for calcium binding: Multiple structural contexts and implications for evolution. J Mol Biol 343(4): 971-984. Residues binding to metal using side chains are in red (direct interaction with calcium) or purple (through-water interaction). Secondary structure as defined by STRIDE [74] is indicated as follows: α-helices, blue shading; β-strands, yellow shading; 3_{10} helices, green shading; turns, brackets.

New folds

```
2z2x
 202 QAILGPDGVADKDGDGIIAGDPDDDAAEVISMSL 235

2zxq
 591 LPQMAVAIAGDENEDGAVNWQDGAIAYRDIMNNP 624

3c68
 421 AYHDWWLRNRDHNGNGVPEYGATRDKAHNTESGE 454....544 YSLLQESVDQA 554

3km5
1169 GEAPAEWTTIDADGDGQGWLCLSSGQLDWLTAHG 1202..1216 ALNPDNYLISK 1226
```

Calcium blades

```
1jv2
 274 AYFGFSVAATDINGDDYADVFIGAPLFMDRGSDG 307

2z8r
 148 TYSANDASVGDVDGDGQYELILKWDPSNSKDNSQ 181

3hx6
 841 PNGLSSPRLADNNSDGVADYAYAGDLQGNLWRFD 874

2bwr
 167 RLDRHLRFLADVTGDGLLDVVGFGENQVYIARNS 200
```

From Rigden & Galperin (2004)

```
1exr
 10 AEFKEAFALFDKDGDGTITTKELGTVMRSLGQNP  43

1gcg
124 AKHWQANQGWDLNKDGKIQYVLLKGEPGHPDAEA 157...200 NANKIEVVIAN 210

1kwh
161 YTVLKAFKEKDPNGNGKADEVPFIDRHPDEVFRL 184
```

1daq
1 ___MSTKLYGDVNDDGKVNSTDAVALKRYVLRSG 21

1qut
227 MPSSYKQYAVDFSGDGHINLWDPVDAIGSVANYF 260

1acc
167 PELKQKSSVPDRDNDGIPDSLEVEGYTVDVKNKR 200

1lwj
3 GYQIYVRSFRDGNLDGVGDFRGLKNAVSYLKELG 36

1h71
39 QLTRSGASWHDLNNDGVINLTYTFLTAPPVGYAS 72...109 AARGDDGHQTF 119

1kap
436 AASKAGSLAIDFSGDAHADFAINLIGQATQADIV 469

1ux6 binuclear
833 RIGDTCDNNQDIDEDGHQNNLDNCPYVPNANQAD 866

1ux6 mononuclear
892 CRLVPNPDQKDSDGDGRGDACKDDFDHDSVPDID 925

1vjj
291 RVITNFNSAHDTDRNLSVDVYYDPMGNPLDKGSD 324
