# Supplementary figures and images for: New Structural and Functional Contexts of the Dx[DN]xDG Linear Motif: Insights into Evolution of Calcium-Binding Proteins
Source: PLoS One. 2011 Jun 24;6(6):e21507. doi: 10.1371/journal.pone.0021507 (PMC3123361; doi:10.1371/journal.pone.0021507)

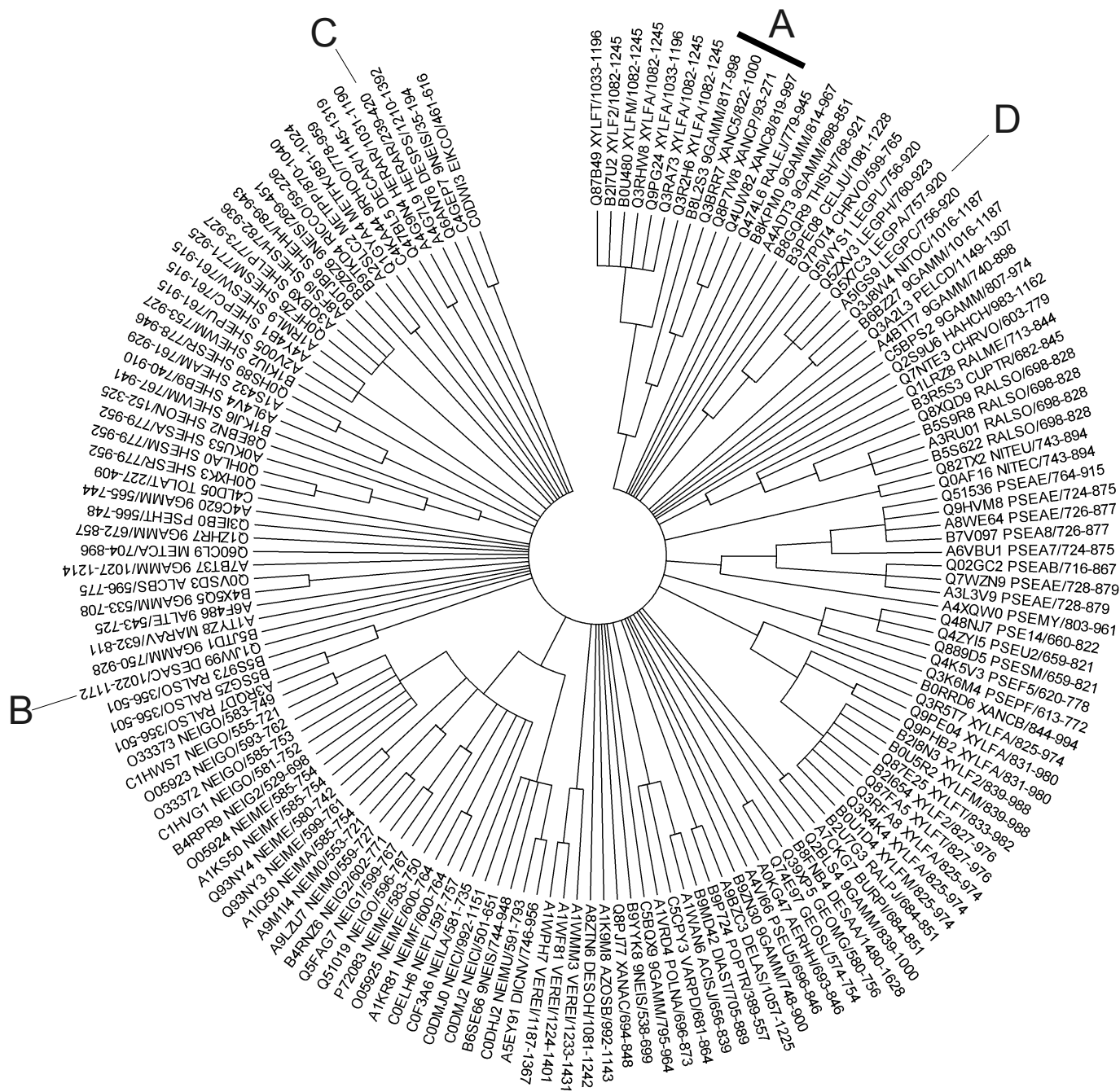

Supplement: Figure S2 — Bootstrapped, neighbour-joining tree made with MEGA4 [90] using sequences edited and realigned from Pfam entry PF05567. Nodes with less than 50% bootstrap support have been collapsed. Individual sequences and groups mentioned in the text are labelled as follows: A, PilY1 sequences from Xanthomonas campestris and Stenotrophomonas sp.; B, Desulfuromonas acetoxidans PilY1-like protein Dace_0383 (UniProt: Q1JW99); C, Herminiimonas arsenicoxydans protein HEAR2375 (UniProt: A4G7L9); D, Legionella pneumophila protein Lpp0682 (UniProt: Q5X7C3). (PDF) [file pone.0021507.s002.pdf]
