# Supplementary material for: New Structural and Functional Contexts of the Dx[DN]xDG Linear Motif: Insights into Evolution of Calcium-Binding Proteins
Source: PLoS One. 2011 Jun 24;6(6):e21507. doi: 10.1371/journal.pone.0021507 (PMC3123361; doi:10.1371/journal.pone.0021507)
Supplement: Table S1 — Families containing Dx[DN]xDG calcium-binding loops, including those in [15]. (PDF) [file pone.0021507.s003.pdf]

Table S1: Families containing Dx[DN]xDG calcium-binding loops including those described in Rigden & Galperin (2004) The DxDxDG motif for calcium binding: Multiple structural contexts and implications for evolution. J Mol Biol 343(4): 971-984.

	Representative[a]	SCOP class; fold of domain containing Dx[DN]xDG loop	PDB code, reference; position(s) of the first D of Dx[DN]xDG	Distribution of proteins containing Dx[DN]xDG loop	Frequency of Dx[DN]xDG loop in homologous proteins[b]	R.m.s. fit of Dx[DN]xDG to the first calmodulin motif	Distance between Dx[DN]xDG and later Ca^{2+} ligands (residues)	Function of bound calcium
**Novel structural contexts**								
	Thermococcus kodakaraensis subtilisin	α+β; Subtilisin-like[b]	2z2x [92]; 212	Some thermophilic archaea	<1% (Pfam PF00082)	0.12 Å	4/7	Folding, in the context of a 25 residues insertion [23]
	Bifidobacterium longum endo-α-N-acetylgalactosaminidase	All β; Supersandwich[b]	2zxq [21]; 601	Bifidobacteria	Approx 7%	0.15 Å	5	Possibly structural [21]
	Escherichia coli YgjK from glycoside hydrolase family GH65	All α; α/α toroid[b]	3c68 [20]; 431	Some γ-proteobacteria	Approx 7% (Pfam PF01204)	0.12 Å	2/112	Not known
	Porphyromonas gingivalis gingipain adhesin domain	All β; Galactose-binding domain-like[c]	3km5 [22]; 1179	*Porphyromonas gingivalis*, *Flavobacterium bacterium*	Approx 90% (Pfam PF07675)	0.35 Å	36	Possibly structural [22]
**Calcium blades**								
	Human integrin αVβ3	All β; 7-bladed β-propeller	1jv2 [93]; 284; 349; 413	Eukaryotic	100% of 3 sites (Pfam PF01839)	0.61-1.07 Å	2	Potentially regulatory [93]
	Bacillus subtilis rhamnogalacturonan lyase	All β; 8-bladed β-propeller[c]	2z8r [24]; 158; 222; 369	[f]	[f]	0.22-0.36 Å	2	Not known (further calcium-binding site required for activity) [24]
	Pseudomonas aeruginosa pilus-biogenesis factor PilY1	All β; 7-bladed β-propeller[c]	3hx6 [25]; 851	Bacterial	Approx 75% (Pfam PF05567)	0.37 Å	2	Regulation of pilus biogenesis and motility [25]
	Psathyrella velutina lectin	All β; 7-bladed β-propeller[c]	2bwr [26]; 177; 233; 343[e]	[f]	[f]	0.48-0.55 Å	2	Possibly structural [26]
**From Rigden and Galperin, 2004**								
	Paramecium tetraurelia calmodulin	All α; EF Hand-like	1exr; 20; 56; 93; 129	Mainly eukaryotic, few bacterial, very few archaeal	Almost 100% of variable number of sites (4 in representative)	0.00 - 0.42 Å	5 (when present)	Regulation or structural or buffering/transport

	Salmonella typhimurium periplasmic galactose-binding protein	α/β; Periplasmic binding protein-like I	1gcg; 134	Bacterial	Approx. 3%	0.27 Å	3/65	Structural
	Sphingomonas sp. periplasmic alginate-binding protein	α/β; Periplasmic binding protein-like II	1kwh; 171	Bacterial	Approx 1%	0.36 Å	2/3	Potentially sensor
	Clostridium thermocellum dockerin	All α; Type I dockerin domain	1daq; 8; 40	Bacterial, very few archaeal	100% of 2 sites	0.98-1.12 Å	5	Structural
	Escherichia coli soluble lytic transglycosylase Slt35	α+β ; Lysozyme-like	1qut; 237	Bacterial	100%	0.42 Å	8	Not known
	Bacillus anthracis protective antigen	Membrane and cell-surface; Anthrax protective antigen	1acc; 177	Bacterial	100%	0.31 Å	2[d]/5	Structural
	Thermotoga maritima 4-α-glucanotransferase	α/β; TIM β/α-barrel	1lwj; 13	Eukaryotic and bacterial	Approx. 35%	0.71 Å	2	Not known
	Pseudomonas 'Tac II 18' alkaline protease	α+β; Zincin-like	1h71; 49	*Pseudomonas, Serratia*	Approx. 35%	0.18 Å	2/59	Not known
	Pseudomonas aeruginosa alkaline protease	All β; β-roll	1kap; 446	*Pseudomonas aeruginosa*	Approx. 15%	0.37 Å	2	Not known
	Human thrombospondin-1	Small proteins; TSP type-3 repeats	1ux6; 828; 843; 866; 879; 915;<br>902	Metazoan	100% of 5 binuclear sites<br>100% of 1 mononuclear site	0.18-0.26 Å<br>0.24 Å	2[c]/5<br>2[c]	Regulation of binding
	Human transglutaminase 3	α+β; Cysteine proteinases	1vjj; 301	Eukaryotic	Approx 20%	0.42 Å	18	Regulation of catalytic activity
	Thermoascus aurantiacus cellulase	α/β; TIM β/α-barrel	1gzj; 287	Bacterial and yeast	Approx. 10%	0.72 Å	No later D or E predicted to bind calcium	Not known

[a] With the exception of the *Thermoascus aurantiacus* cellulase (last line of the Table), all proteins have been demonstrated to bind calcium ions, see Rigden & Galperin (2004) for additional references

[b] As defined by Pfam, SMART or by full-length matches in PSI-BLAST (E-value of 0.0001) run until convergence.

[c] Based on the entry for a homologous protein or from authors' description

[d] Through water interaction with calcium

[e] The motif commencing from residue 233 is not bound to Ca^{2+} in the deposited structure but crystal soak data show that it is capable of doing so [26]

[f] A distinct group could not be defined with PSI-BLAST
